# The Astonishing Behavior of Electric Eels

**DOI:** 10.3389/fnint.2019.00023

**Published:** 2019-07-16

**Authors:** Kenneth C. Catania

**Affiliations:** Department of Biological Sciences, Vanderbilt University, Nashville, TN, United States

**Keywords:** predator, gymnotidae, electrocyte, evolution, electroreception, humboldt, *Electrophorus electricus*

## Abstract

The remarkable physiology of the electric eel (*Electrophorus electricus*) made it one of the first model species in science. It was pivotal for understanding animal electricity in the 1700s, was investigated by Humboldt and Faraday in the 1800s, was leveraged to isolate the acetylcholine receptor in the 20th century, and has inspired the design of new power sources and provided insights to electric organ evolution in the 21st century. And yet few studies have investigated the electric eel’s behavior. This review focuses on a series of recently discovered behaviors that evolved alongside the eel’s extreme physiology. Eels use their high-voltage electric discharge to remotely control prey by transcutaneously activating motor neurons. Hunting eels use this behavior in two different ways. When prey have been detected, eels use high-voltage to cause immobility by inducing sustained, involuntary muscle contractions. On the other hand, when prey are hidden, eels often use brief pulses to induce prey twitch, which causes a water movement detected by the eel’s mechanoreceptors. Once grasped in the eel’s jaws, difficult prey are often subdued by sandwiching them between the two poles (head and tail) of the eel’s powerful electric organ. The resulting concentration of the high-voltage discharge, delivered at high-rates, causes involuntary fatigue in prey muscles. This novel strategy for inactivating muscles is functionally analogous to poisoning the neuromuscular junction with venom. For self-defense, electric eels leap from the water to directly electrify threats, efficiently activating nociceptors to deter their target. The latter behavior supports a legendary account by Alexander von Humboldt who described a battle between electric eels and horses in 1800. Finally, electric eels use high-voltage not only as a weapon, but also to efficiently track fast-moving prey with active electroreception. In conclusion, remarkable behaviors go hand in hand with remarkable physiology.

## Introduction

You might say that electric eels need no introduction. Most people have heard of them and are aware of their unusual ability to generate powerful electrical discharges for offense and defense. But it would probably come as some surprise to many readers that electric eels played a pivotal role in the early development of the science of physiology and their anatomy helped inspire Volta to develop the battery, which he called an artificial electric organ similar to the electric eel’s (Finger and Piccolino, 2011). In the 1700s, when our understanding of electricity was in its infancy and the Leyden jar was the primary device for electrical experiments, the question of whether animals could produce electricity was paramount. Strongly electric fish were well-known, but how their mysterious emissions were produced, and whether this was the same “force” produced by a Leyden jar was a matter of intense debate.

In the early 1770s, evidence in favor of animal electricity was tantalizing but inconclusive. Investigators working with the strongly electric torpedo had established that conductors transmit the torpedo’s emissions but insulators such as wood or wax did not (Wu, 1984). Both fishermen and philosopher-scientists of the time rated the subjective “shock” from a torpedo and a Leyden jar as the same (Piccolino and Bresadola, 2002). Moreover, if a group of people formed a ring holding hands, each would feel the shock from a Torpedo—as occurred for a Leyden jar. Electric fish fell short in one key area–they could not (as yet) produce the all-important “spark” that typified the electric force.

Enter the electric eel. Because the peak electrical potential of a Torpedo (50 volts) is much lower than that of an eel (400–600 volts) it was very difficult to obtain a gap-crossing spark from the former. In 1775, John Walsh experimented with eels and succeeded in demonstrating the spark repeatedly to colleagues and visitors (Piccolino and Bresadola, 2002). It was a pivotal moment in the history of science and kicked off the field of animal physiology.

But the experiments of John Walsh were by no means the end of the scientific community’s obsession with electric eels. Others followed in his footsteps, including Alexander von Humboldt and Michael Faraday. Humboldt reported many details of how and when shocks were conveyed from eels to humans (von Humboldt, 1806) but by far his most famous link to eels was the unconventional way he (supposedly) obtained specimens. While traveling in South America, Humboldt was eager to find eels, but initially could only obtain animals that had been poisoned, and these were useless for study. He eventually succeeded by hiring fishermen who told him they would “fish with horses.” As the story goes (von Humboldt, 1807) the fishermen herded about 30 horses and mules into a pool containing eels, and a spectacular battle ensued. The horses were contained within the pool by the fishermen, and the eels attacked. Two horses died within 5 min, and others managed to escape and collapsed on the ground next to the pond. Humboldt thought all the remaining horses would be killed, but before this could happen the eels were exhausted. This was apparently the point of the exercise, as the fishermen were then able to safely collect five specimens for Humboldt. Not everyone believed his story, but it is now supported by recent discoveries that will be reviewed here.

Faraday’s much later foray into eel research included a meticulous investigation of the many parallels between electrical phenomena and eel discharges. His results, coming from perhaps the world’s foremost electrician of the time, shored up the belief in animal electricity. He also published several prescient observations of eel behavior with interpretations that are, as in the case of Humboldt’s reports, supported by recent investigations (Faraday, 1838).

Despite the centuries-long scientific interest in electric eels, there is still much to learn from this species. What follows is a summary of the author’s recent work investigating electric eel behavior and the effects of its electric organ discharge (EOD) on nearby animals. This research began simply as a photography project but turned into a multi-year scientific investigation.

## New Insights Into EOD Function

### Electric Eels Have Two Forms of EOD

**Figure 1:**
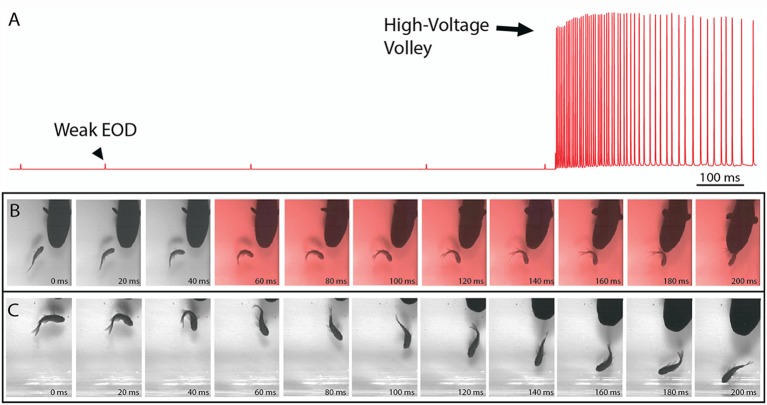
The electric organ discharge (EOD) and its effect on prey. **(A)** Recording showing the two different EODs of an electric eel. Each is a monophasic, head-positive pulse lasting approximately 1 ms. The low voltage output (arrowhead) occurs at a low rate. The high voltage output (arrow) is much stronger and occurs at a much higher rate in volleys of up to 500 Hz during the predatory strike. High-voltage volleys are also used for defense. **(B)** An approaching eel elicits a C-start escape response in a goldfish, but the goldfish is immobilized by a volley of high-voltage pulses (red frames) and captured within 200 ms. **(C)** A similar encounter during which the fish was not immobilized and thus rapidly outpaced the eel. In the final frame (far right) this distance between eel and fish has increased and the prey velocity is greater than the eel velocity (from Catania, 2015a, reproduced with permission).

Electric eels provide an informative example of strongly electric fish because they uniquely emit two different types of EOD (Coates et al., 1940; Bullock, 1969; Bauer, 1979). Each is of the same form, consisting of a roughly 1 ms, monophasic pulse, but one is far stronger than the other. Figure 1 illustrates these two different outputs in a single trace from a behaving eel—recorded from electrodes in the aquarium. The low-voltage output comes at a low rate in a resting eel but may be emitted at 10–20 Hz when the eel is excited and hunting (Bauer, 1979). The high-voltage EOD is far stronger and is emitted at rates of up to 500 Hz. These two different EOD’s have been thought to provide extant examples for two different functions: low-voltage discharges used for active electroreception (and perhaps communication) and high-voltage discharges used as a weapon. As the reader will see below, recent studies shown there is more to this story.

The mechanism by which electric eels generate either a weak EOD or a strong EOD has been determined in some detail (Bennett, 1968, 1970). Given the similar form of the two outputs, it is perhaps not surprising that the weak EOD is emitted by simply activating a subset of the eel’s electrocytes (the eel’s electrocytes are divided among three different electric organs which are referred to here as the eel’s electric organ for simplicity). Surprisingly, an action potential is sent to every electrocyte in the eel’s body when the weak EOD is emitted. But for the majority of electrocytes, the excitatory post-synaptic potentials are sub-threshold and do not result in an electrocyte action potential. Thus the weak EOD is the result of a subset of low-threshold electrocytes that can be activated by a single motor neuron action potential. The high-voltage EOD is emitted simply by sending a very high rate of action potentials to all of the same electrocytes. This, in turn, results in temporal summation in the higher-threshold electrocytes such that every electrocyte in the eel’s body is activated. As a result of this simple mechanism for generating EODs of two different strengths, every high-voltage volley is (necessarily) immediately preceded by a single low-voltage, weak EOD (Bauer, 1979).

### Eel High-Voltage EOD’s Temporarily Immobilize Prey

Recent high-speed video recordings of electric eels hunting revealed an unusual reaction of prey fish electrified by high-voltage volleys (Catania, 2014). Within 3–4 ms of the first EOD in the volley, all prey voluntary movement was arrested and the fish floated “statuesque” with fins and body immobile throughout the volley (the fish was invariably captured by the eel shortly thereafter). Even when fish were in the midst of a rapid escape response and bent into a C-shape, the high-voltage froze all subsequent body movements. This was surprising because it is easy to imagine high-voltage electrical impulses inducing some form of movement, rather than immobility. When the eel’s strike missed the fish and the high-voltage volley was discontinued, most fish immediately resumed their escape. Thus the prey were usually not killed or disabled.

**Figure 2:**
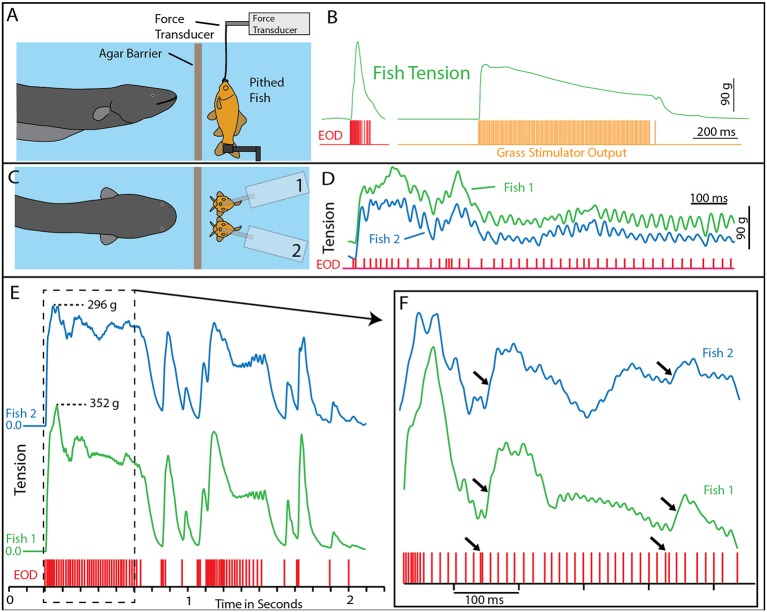
Paradigm and results for eel-induced muscle tension measurements in prey. **(A)** To measure prey muscle tension, a pithed fish was attached to a force transducer while an eel (behind and agar barrier) was fed earthworms (which it shocked with high-voltage volleys). **(B)** Onset of fish tension (green) occurred in roughly 3 ms and was generally similar to the maximum whole-body fish tension that could be induced through direct stimulation (orange trace) with an SD9 Grass stimulator. **(C)** To compare responses of two different fish under various conditions (see Catania, 2014), two force transducers were placed side by side. **(D)** The dual fish paradigm unexpectedly revealed that long eel interpulse intervals result in nearly identical patterns (green and blue traces) of individual twitches in the two, adjacent fish. **(E)** Overall tension responses in two fish were also similar at a more compressed time scale (blue and green traces, different cases from “**D**”). **(F)** An expanded time scale shows the marked effect of eel doublets-closely spaced EODs (arrows) on corresponding fish tension. This suggests that doublets have the same strong tension-inducing effect in fish as shown for other experimental preparations (from Catania, 2015a, reproduced with permission).

A potential explanation for this effect was the induction of muscle contraction by the EOD, in a manner analogous to a law-enforcement TASER. This possibility was investigated using a whole-fish preparation immersed in the aquarium with the eel but separated by an electrically permeable agarose barrier (Kalmijn, 1971). Figure 2 illustrates the preparation that was used. The fish was first anesthetized and pithed to destroy the brain, and the hole was sealed with cyanoacrylate. This preparation, with muscles that remained viable throughout the experiment, was then attached to a force-transducer positioned above the water. High-voltage volleys were easily and repeatedly elicited from the nearby eel simply by feeding it earthworms in the adjacent chamber.

The results of this experiment showed that eels induce massive whole-body muscle contractions (Figure 2). By comparing eel induced contractions to those induced by direct stimulation with a Grass SD9 stimulator, it was shown that eels induce massive whole body tension, similar to that induced by a stimulator with leads directly connected to the fish body. Presumably, the statuesque appearance of prey during the eel’s volley results from simultaneous contraction of equally powerful trunk muscles on both sides of the fish. The importance of this ability is evident when a successfully escaping fish track is compared to that of an eel-immobilized fish during the attack (Figures 1B,C). It is usually obvious that an active fish would have escaped (see also Catania, 2014, 2015a). This is not to say that eel strikes are slow, rather escaping fish are fast.

### Eels Cause Muscle Tension by Remotely Activating Prey Motor Neurons

The discovering that electric eel high-voltage volleys cause powerful whole-body muscle contractions in nearby prey immediately raised a follow-up question: what was the mechanism by which muscles were activated? The two most likely possibilities were either the direct depolarization of the prey’s muscles or alternatively, activation of the associated motor neurons. This question was addressed using two side-by-side fish preparations attached to force transducers, such that one served as a control and the other could be pharmacologically manipulated (Figure 2). When one preparation was injected with curare to block the neuromuscular junction, and the other sham injected, the muscle contractions in response to eel volleys were eliminated in the former but not in the latter (Catania, 2014). This demonstrated that high-voltage volleys were not directly depolarizing prey muscles. The experiment was then extended by pithing the spinal cord of the fish (double-pithing). There was no difference in latency or tension magnitude in double-pithed vs. brain-pithed fish, indicating the spinal cord is not necessary for the fish muscle response. These experiments showed that electric eels immobilize prey by remotely activating the peripheral branches of motor neurons.

As often happens, the experiments described above also revealed unanticipated details about the mechanism. The electric eels used to activate the fish preparation were repeatedly fed earthworms in order to elicit many high-voltage volleys. Over time some eels apparently became fatigued, because the rate of their high-voltage EOD slowed and the interpulse interval became variable. In these cases, individual twitches emerged on the tension traces in the nearby experimental fish preparations. Moreover, in the side-by-side fish preparations, the twitch responses were nearly identical (Figures 2E,F). These results indicated that each high-voltage EOD from the electric eel typically results in an action potential in the motor neurons of nearby prey. High rates of the EOD result in fused muscle tension, whereas lower rates reveal individual twitches—as would be observed in a muscle physiology laboratory.

The implications of these results are remarkable. Ultimately, the motor neurons in the electric eel are activating the muscles of a nearby animal in a one-to-one fashion. By amplifying the “signal” from its own motor neuron, the eel’s electrocytes provide a mechanism for remotely controlling another animal. In a sense, the eel’s high-voltage discharge can be viewed as an action potential traveling through the water, destined to activate the motor neurons in any nearby animal.

Though astonishing, in retrospect these results might have been predicted from the analogous mechanism underlying the function of a law-enforcement TASER, or the mechanism of more commonly used transcutaneous electrical nerve stimulation paradigms (TENS) used for human muscle therapy (Sweeney, 2009).

**Figure 3:**
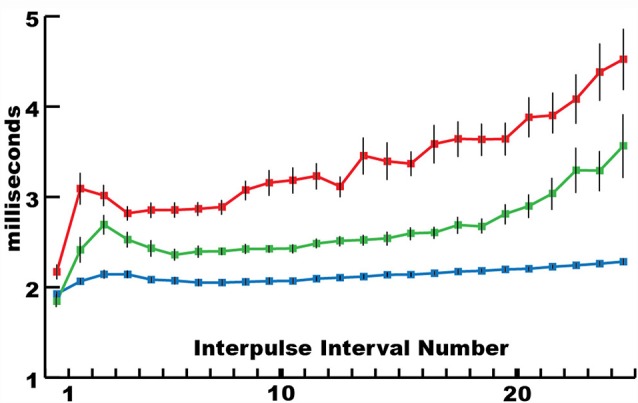
The mean interpulse intervals for differently sized electric eels. The longest interpulse intervals (red) corresponded to the smallest eel (50 cm), intervals of intermediate length corresponded to the intermediate eel (approximately 75 cm), whereas the shortest intervals (blue) corresponded to the largest eel (115 cm). For each specimen, the first interpulse interval was the shortest. Bars are standard error. See Catania (2014) for additional statistics (from Catania, 2015a, reproduced with permission).

This mechanism for incapacitating prey suggested that new insights might be gained by considering the eel’s volley from the perspective of prey motor neurons. For example, Figure 3 shows the average interpulse interval for the high-voltage volleys of three different electric eels. In each case, a significantly (statistically) shorter interpulse interval was found for the first two discharges in the volley. As it turns out, numerous investigations of neuromuscular systems have found that the rate of muscle contraction can be maximized by including two closely spaced action potentials at the beginning of the motor neuron train. These are called doublets (Celichowski and Grottel, 1998; Cheng et al., 2013; Pedersen et al., 2013). More detailed investigation of the optimal motor neuron train for maximizing muscle tension (Zajac and Young, 1980a, b) reveals a pattern of action potentials that is similar in form to the first part of an electric eel’s volley. This raises the possibility that eel volleys have been specifically selected to most efficiently induce rapid and powerful muscle contractions in nearby animals, and hence to most rapidly immobilize animals that might otherwise escape.

Alternatively, because eel electrocytes are derived from muscles and innervated by motor neurons, the eel’s motor neuron output (and therefore its EOD) might be constrained in a manner similar to that of a wide range of neuromuscular systems. Put another way, the similarity between the beginning of an eel’s EOD and the optimal motor neuron train found for maximal muscle activation could reflect a constraint on both systems at peak power output. And yet this seems unlikely, given the incredible variation in the form and rate of EOD’s exhibited by a diversity of electric fish. Moreover, electric eels have another way of remotely controlling prey that also seems to make use of an optimal strategy.

### Eels Emit High-Voltage Doublets to Induce Movement in Hidden Prey

In 1979 the results of Bauer’s investigation of electric eel hunting behavior and EOD were posthumously published (Bauer, 1979). He reported that: “Introduction of prey into the aquarium arouses the eel, causing it to swim around, but often stopping in a particular corner of the aquarium. During these stops, two high-voltage pulses with an interval of about 2 ms are emitted.” He reported this behavior as typical of hunting eels.

**Figure 4:**
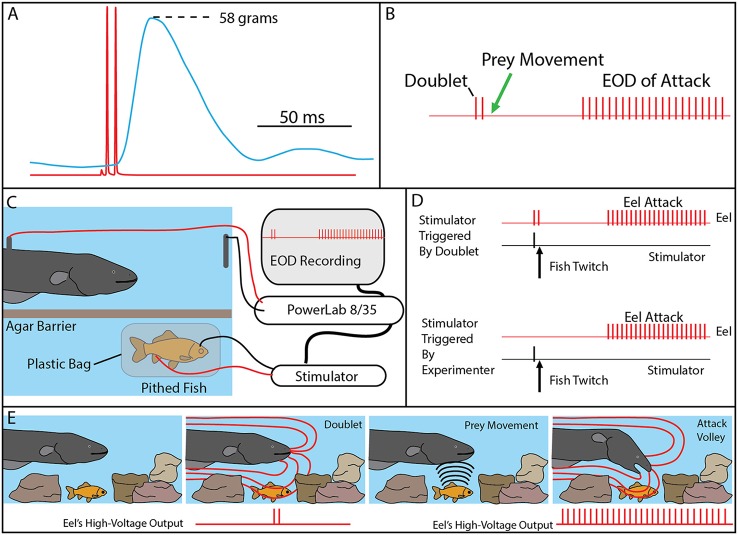
The use of doublets in eel predatory behavior. **(A)** Example of an isolated doublet (red) inducing strong tension (blue) in a nearby pithed fish attached to a force transducer. **(B)** Schematic of the doublet output followed by prey movement (twitch) and then a full high-voltage volley. **(C)** Schematic of the paradigm used to investigate the use of doublets during hunting. A pithed fish was enclosed in a plastic bag, while connected to an SD9 Grass stimulator that could induce twitch when the eel emitted a doublet. **(D)** The doublet was followed by a full volley (and predatory strike) if twitch was immediately triggered through the stimulator (upper trace), but no attacks were elicited in the absence of prey twitch (not shown). In the absence of doublets, full volleys and strikes could be elicited by randomly generated fish twitch (bottom). **(E)** Schematic illustration of the use of doublets to detect prey in normal hunting behavior (from Catania, 2015a, reproduced with permission).

Bauer’s observations took on new significance in light of the mechanisms described above, by which eels activate motor neurons in nearby prey with each high-voltage discharge (Catania, 2014). This is especially true given that doublets at the beginning of a motor neuron action potential train are particularly efficient and producing powerful muscle contractions. All of the eels used in the recent studies (reviewed here) gave off doublets while hunting, and it was a frequent behavior during the fish muscle-tension experiments described above. As would be predicted, the doublets resulted in a massive whole-body twitch in the nearby fish preparations (Figure 4A). In some cases, after giving off a doublet the eel tried to break through the thin agarose barrier (which was reinforced with nylon netting) to reach the fish.

The context during which doublets were emitted, and the preliminary behavioral observations, suggested that doublets might function by causing prey movement that is detected by the eel’s neuromasts (mechanoreceptors). Electric eels are extremely sensitive to water movements and often respond with a high-voltage volley and strike. In addition, eels hunting live prey in a complex environment (or when prey were under an agarose barrier) sometimes gave off a doublet, resulting in prey twitch, followed (20–40 ms later) by the eel’s full, tetanus inducing high-voltage volley and suction feeding strike (Figure 4B).

Determining whether eels use doublets to detect induced prey movements required a paradigm in which the prey’s response was under the control of the experimenter. This was achieved using a variation of the pithed fish preparation (Figures 4C,D). In this case, a stimulator was connected to the fish, and the preparation was sealed in a plastic bag such that the fish preparation was electrically isolated from the eel. The stimulator was then controlled through a data acquisition unit that monitored the eel’s EOD, such that fish twitch could be triggered in response to a doublet (or not triggered, at the discretion of the investigator).

The first question to be addressed was the latency of the eel’s response to fish twitch. If eel’s were responding to fish twitch in their natural doublet-hunting behavior, then their reaction time would have to be in the 20–40 ms range. This was, in fact, found to be the case. When the eels were close to the fish preparation and the investigator trigger the stimulator, eels responded to the twitch with a high-voltage volley and strike toward the preparation with a delay of 20–40 ms.

When eels gave off doublets near the fish preparation, but no fish twitch was triggered, no attack was elicited. Moreover, eels never gave off doublets, followed by a full volley, in the absence of fish twitch. However, the key experiment was to configure the data acquisition unit to immediately trigger fish twitch when the eel gave of a doublet. When this was arranged, the natural doublet-hunting behavior was recreated (Figure 4). Eels gave off a doublet, the fish twitched (as a result of the EOD triggered stimulator) and then the eels attacked with a full volley and strike toward the fish preparation. A number of control experiments confirmed that eels were not responding to visual cues from the moving fish or electrical impulses from the stimulator leads (Catania, 2014).

The remarkable conclusion from these experiments is that eels have dual modes of prey remote control. When a nearby fish is detected, a full volley of high-voltage impulses causes rapid and powerful muscle contractions preventing escape. When prey are hidden, or their identity is uncertain, eels can induce involuntary twitch, revealing their approximate location (Figure 4E). In essence, the doublet allows the eel to ask the question of a nearby object: are you alive? Prey have no choice but to respond.

It has been previously suggested, that both strongly electric catfish (Belbenoit et al., 1979) and the strongly electric torpedo might use this kind of hunting strategy as well (Belbenoit and Bauer, 1972). The former suggestion was inferred from recordings of catfish hunting in the wild, during which some volleys were preceded by brief pre-volleys. It is astonishing, however, that Michael Faraday (using only his hands) inferred the electric eel’s ability to detect and attack EOD induced movement in 1838. His description is so prescient as to seem incredible, especially given the limitations of his equipment. I quote his comments in full here: “*The Gymnotus appears to be sensible when he has shocked an animal, being made conscious of it, probably, by the mechanical impulses he receives, caused by the spasms into which he is thrown*” (Faraday, 1838).

### Active Electroreception by Electric Eels

As described above, electric eels have both a low voltage and high voltage EOD. Undoubtedly the first observation of active electroreception comes from Walsh’s experiments on electric eels in the 1770s (see Wu, 1984). Walsh noticed that when two wires were put into the water with the electric eel and extended some distance from the container, the eel was able to detect when the two wires were connected. The eel responded by giving of its high voltage volley.

At the time, no one was aware of the low voltage EOD that is constantly emitted as eels explore their surroundings (which was almost certainly the basis for the eel’s ability). The explanation was not available until Lissmann (1958) showed that weakly electric fish use low-voltage EOD’s for active electroreception. Not long after Lissmann’s discovery, Hagiwara et al. (1965) investigated the physiological properties of the eel’s electroreceptors and concluded that the low-voltage EOD was, in fact, used for active electroreception (see also Keynes and Martins-Ferreira, 1953).

Lissmann’s discovery of active electroreception provided the missing, functional intermediate needed to explain the evolution of strongly electric species. Thus for electric eels, the evolutionary trajectory was easy to envision. Their ancestors presumably used an electric organ for navigation, and this was progressively enlarged to provide an electrical weapon (as previously noted, the eel’s electrocytes are actually divided among three separate organs). The retention of the low-voltage EOD for active electrolocation seemed to fit well with such a functional bifurcation: low voltage for electrolocation and high-voltage for offense and defense. In the author’s view (prior to 2015) a remaining question was how the eel’s sensitive electroreceptors dealt with the high-voltage volleys, the presumption being that the electric sense was shut down completely during the high-voltage EOD. Recent data show this is not the case.

### The Use of High-Voltage for Active Electroreception

Recall the electric eels did not grasp the electrically insulated prey in the course of doublet hunting experiments described above. Instead, the strikes were aborted without a final “bite.” This was obvious because electric eels are air-breathers and they hold air in their mouths between breaths. As a result, their suction feeding is accompanied by sudden expulsion of air from the operculum. This was fortuitous because it made the absence of the final component of the strike more obvious during experiments. Before describing the next experiments, it is import to re-emphasize that electric eel predatory strikes occur in conjunction with the high-voltage volley; there are no low-voltage EOD’s emitted during the strike. Thus evidence of ongoing active electroreception during the strike must be attributed to the high-voltage EOD.

As a preliminary test for the possibility that electric eels were using the high-voltage EOD for active electroreception, a conductive carbon rod was placed next to the electrically isolated fish preparation (to interpret this experiment it is important to note that prey are conductors, thus the carbon rod was a “stand-in” for a prey item). The fish was made to twitch by activating the stimulator and the eel then gave off its high-voltage volley and struck toward the electrically isolated fish as previously described. But this time, the eel altered course, moved over the conductor, broke through the agarose barrier, and sucked the conductive rod into its jaws (see movies in Catania, 2015b). This dramatically different response in the presence of a conductor suggested the eels depend on the high-voltage EOD to guide their strikes during normal predatory behavior.

**Figure 5:**
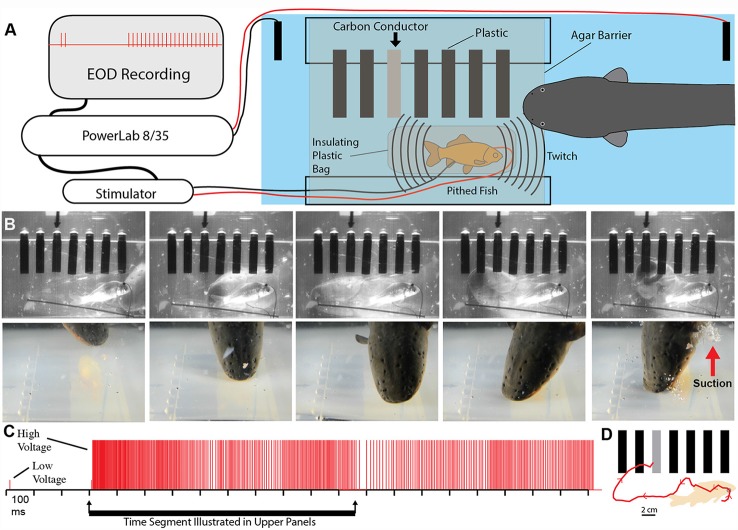
Paradigm showing that electric eels find and attack conductors. **(A)** Recording and stimulator configuration that triggered pithed-fish twitch and eel attack in the presence of six plastic rods and one conductor (arrow). **(B)** Plates from high-speed video (top) and real-time (bottom) of same trial illustrating circuitous path to conductor. **(C)** Eel low and high voltage discharge marked with short, and tall ticks respectively, illustrating the exclusive use of high-voltage during strike movement. **(D)** Eel path to conductor (from Catania, 2015b, reproduced with permission).

To test this possibility more rigorously, a number of additional experiments were devised. The first was an elaboration of the carbon rod paradigm. An apparatus was made that could hold seven different rods of similar shape and appearance (Figure 5). One rod was a carbon conductor (imitation prey), and the other six rods were plastic non-conductors. The pithed fish preparation (electrically insulated in a plastic bag) was then placed below the carbon rods, and the entire apparatus was covered with a thin agarose barrier that did not block mechanosensory cues. Under these conditions, fish twitch could be generated either by the experimenter triggering the stimulator or by having the data acquisition unit trigger the stimulator in response to a doublet. Electric eels responded to fish twitch with a high voltage volley and strike. In each case, the strike was guided, often on a circuitous path, to the conductor, which was then violently attacked with a suction feeding bite (this is a rapid series of events analyzed in slow motion from high-speed video).

**Figure 6:**
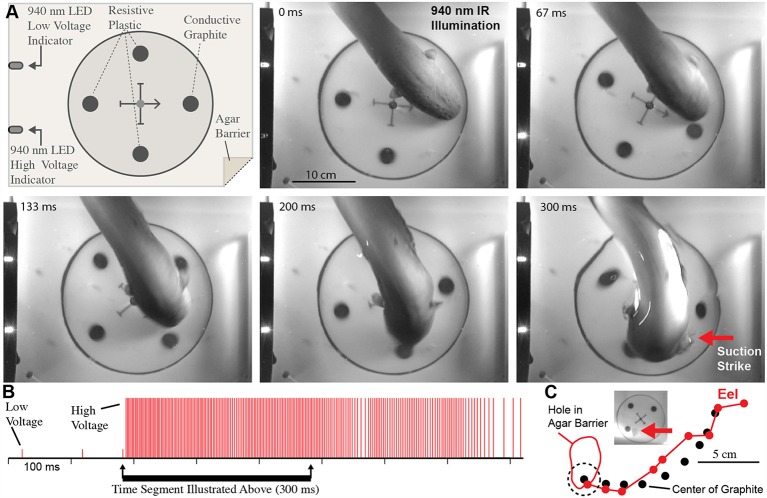
Eel conductor tracking under 940 nm IR illumination. **(A)** Schematic of paradigm and plates showing eel tracking behavior and suction feeding strike to conductor. **(B)** Eel low and high voltage discharge marked with short, and tall ticks respectively, illustrating the exclusive use of high-voltage during strike movement. **(C)** Eel track relative to conductor movement. Inset shows hole in agar (arrow) that was directly over conductor at suction onset (from Catania, 2015b, reproduced with permission).

These experiments seemed to confirm that electric eels use their high-voltage EOD for active electroreception. However, the conclusion is somewhat extraordinary, and therefore additional experiments were conducted to provide the clearest evidence possible (Catania, 2015b). For these additional experiments, a small (2.5 cm diameter) carbon disk was inserted into a larger (16.5 cm diameter) disk that was mechanically driven to spin below an agarose barrier (Figure 6). Three non-conductive plastic disks of the same diameter and appearance were also inserted into the larger spinning disk as control stimuli. The apparatus was illuminated by invisible, 940 nm infrared light, and 940 nm infrared diodes (controlled through a data acquisition unit) were configured to indicated each low voltage EOD and each high-voltage EOD. This paradigm provided redundant control for vision and prevented contact and chemical cues by virtue of the electrically permeable agarose barrier. The eels attacked the moving conductor (the imitation prey) with suction feeding strikes (after initial attacks, eels were rewarded after each strike to maintain the behavior). The results of these and additional experiments clearly showed that electric eels can rapidly track conductors moving on a curved trajectory using their high-voltage EOD. Moreover, the eel’s were able to track the conductors at a greater speed than has been previously reported for active electroreception in other species (Maciver et al., 2001).

Details of conductor tracking indicate that active electroreception using high-voltage is integral to the strike. In retrospect, it seems obvious that some form of sensory feedback is necessary for eels to accurately strike. Although the high voltage EOD prevents prey muscle movement, it does not prevent the continued motion of a fast-moving fish through the water after voluntary behavior has been arrested. In addition, the explosive movement of the eel’s head through the water toward prey causes much additional water motion. As a result, prey are often fast-moving targets, even after their muscles have been inactivated. Finally, it is unlikely that a brief, distant water movement caused by prey—which often triggers the eel’s strike—provides the necessary positional information for an accurate attack. Active electroreception during the strike solves these problems for the eel.

### A Revision of the Evolutionary Trajectory

It is worth recounting Darwin’s discussion of electric fish under the section in the Origin of Species that dealt with difficulties of the theory (Darwin, 1873). Electric fish were considered a problem, in part, because there was no obvious use for the small electric organs that were intermediate between muscles and the large electric organs of eels and rays. The use of the latter for offense and defense was clear, but bridging the “functional gap” between muscle contraction and high-voltage weapons was problematic. Lissmann’s discovery of active electroreception in weakly electric fish seemed to fill in this part of the evolutionary puzzle (Lissmann, 1958). But the present results indicate there was more to the story for eels. Active electroreception using high-voltage shows that, in the case of eels, the electric organ did not simply transition from a sensory role to a weapon. Rather, it most likely added the role as a weapon while retaining its sensory function throughout.

The stages of this evolutionary process are of course lost to history. But it is intriguing to consider, in addition to its role as a weapon, the possibility of the eel’s high-voltage being important for sexual selection (courtship), territoriality, or communication. Assunção and Schwassmann (1995) were able to identify nests of breeding eels and found these were built by males and subsequently defended. Although they did not observe possible courtship or territoriality, it seems an interesting possibility to explore in future in the context of the EOD.

## Dealing With Difficult Prey

### The Dipole Attack

So far the predatory behavior of electric eels has been described and illustrated as it typically occurs when feeder goldfish are provided to an eel in an aquarium. But electric eels live in the Amazon, which includes a wide diversity of fish species and other potential prey. Surprisingly little is known about the diets of electric eels in their natural habitat, but it is obvious that feeder goldfish are not their most challenging prey. Moreover, juvenile eels have much smaller, weaker electric organs and can have difficulty handling even small fish.

**Figure 7:**
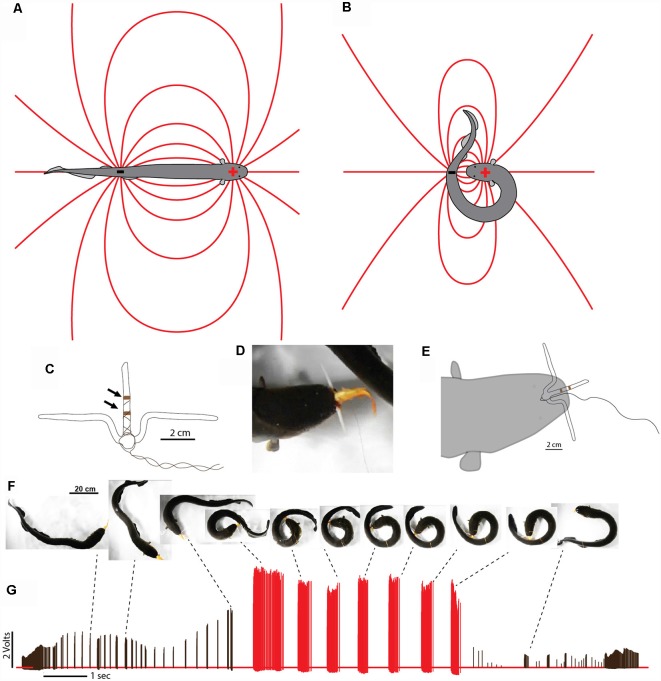
Dipole field and dipole attack. **(A)** Schematic illustration of a dipole field surrounding an electric eel and its change in configuration **(B)** after the eel has brought the two poles close together. Lines indicate electric field lines (a positive test charge would experience a force tangent to the line at any point—in the direction of the negative pole). **(C)** Schematic illustration of electrodes with un-insulated wire (arrows) approximately 1 cm apart. **(D)** View of eel holding electrode-fish preparation tightly. **(E)** Schematic of electrode position during trial. **(F)** Large eel presented with the pithed fish with electrodes. After capture, the experimenter manually jiggled the wire to simulate prey struggling and the eel curled to deliver multiple discharges. **(G)** Voltages recorded from the electrode at different points during the eel’s attack. Black tick marks indicate discharges while “uncurled.” Red tick marks were all recorded while curled. Note the dramatic increase in recorded voltage, and discharge frequency, during the curl relative to the uncurled configuration (from Catania, 2015c, reproduced with permission).

In cases where difficult prey are encountered, electric eels are uniquely suited for a strategy that increases the intensity of their attack. This is because, unlike strongly electric rays or catfish, the eel’s electric organ is linear and extends through its long, thin body. This means the positive and negative poles (the head and tail, respectively) are widely separated in space. During a typical predatory attack, the eel’s electrical discharge forms a (roughly) dipole field around the eel. The positive pole is the region around the eel’s head and the negative poll is the region around the tail (Figures 7A,B). A fish near the eel’s head experiences the effect of the positive pole and almost no effect from the more distant negative pole around the tail. In fact, for a fish situated directly in front of an eel—when the eel’s body is straight—the effect of the negative pole (tail) would subtract from the effect of the positive pole, reducing the strength of the local field.

This would change drastically, however, if the eel were to curl and bring its tail behind and close to the prey. In such a case, the effect of the tail (the negative pole) would be additive (because the prey would be sandwiched between the two poles) and strong (because the negative pole would be close), rather than subtractive and weak. The theoretical effect of such a curling move would be to double the intensity of the electric field experienced by prey, at virtually no cost to the eel.

In fact, electric eels commonly engage in this curling behavior when handling difficult prey (Catania, 2015c). Juvenile eels frequently curl when attacking any prey item, whereas adults curl when handling difficult, struggling prey, or when they have captured a fish that is being held precariously and might otherwise escape. Although the basic physics of dipole fields predict the effect of this curling behavior, a number of experiments were conducted to directly measure the resulting electric field and its effect on prey (Catania, 2015c).

### Measuring Field Strength During the Eel’s Curling Behavior

Measuring the changing intensity of an electric field experienced by prey during an eel’s attack would seem daunting. The common method of monitoring electric fish EOD’s, with electrodes stationary in the aquarium, cannot provide data about the local field strength in and around prey. Nor can an investigator chase a hunting eel with electrodes and hope to get useful data. This problem was solved by leveraging the eel’s aggressive predatory attack.

To measure the electric field within prey, the pithed fish preparation was again used. However, in this case, the fish was impaled on a custom-made, plastic electrode holder (Figure 7C). The recording electrodes consisted of two wrappings of thin copper wire spaced 1 cm apart on the long projection of the T-shaped electrode holder. The thin insulated leads from the electrodes led to a data acquisition unit that recorded the electrical potential. At the same time, the insulated leads provided a convenient handle—much like a fishing line—that could be manipulated by the investigator. Finally, the upper part of the T-shaped electrode holder prevented the eel from swallowing the preparation.

When this preparation was introduced to a hungry eel, it was attacked, sucked into the eel’s mouth, and gripped very tightly (Figures 7D,E). This condition likely mimics natural situations during which prey fish with defensive spines have been caught but are difficult to swallow. By manually vibrating the electrode leads, the investigator was able to imitate struggling by the pithed fish-electrode preparation, and this elicited the eel’s curling behavior.

The preparation provided data from numerous eels, showing that the intensity of the electric field experienced by prey often more than doubled when the eel curled (Figures 7F,G). Recall, that electric eels cannot increase the magnitude of their total power output during the high-voltage volleys, rather every electrocyte is active during each high-voltage EOD (see above). Therefore, the increase in measured field strength resulted from the reconfiguration of the electric field. The electric field was concentrated, so-to-speak, through the prey item, much like focusing the fixed power of a flashlight into a smaller area.

It might seem surprising that, in many cases, the field strength within prey more than doubled when the eel curled. This likely occurred because the tail, containing the negative pole, can be brought very close to the prey (essentially touching), whereas the positive pole of the electric organ is situated at some distance behind the front of the eel’s head (to make room for the eel’s internal organs). Therefore the negative pole (with an effect that is added to the positive pole) may have a greater effect, based on proximity, than the positive pole during the curling behavior.

**Figure 8:**
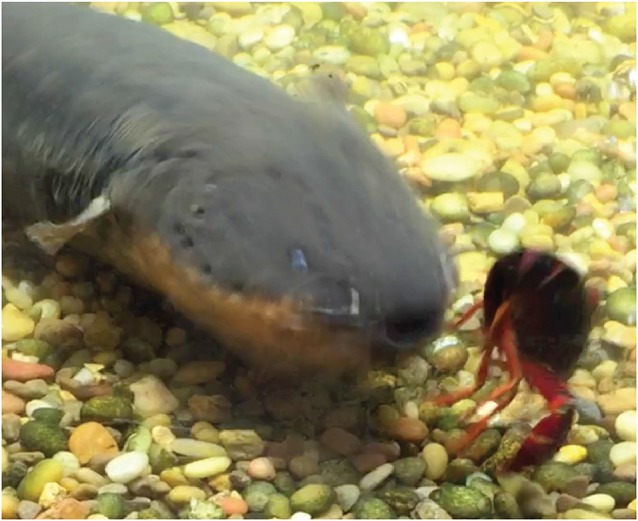
Frame captured from video showing an eel attacking a crayfish. Note that despite the eel’s comparatively large size and ability to cause tetanus in most fish with its high- voltage discharge, the crayfish escape response was not canceled. The appropriate form of the lateral giant escape from the rear-ward attack indicates that the crayfish movement was not caused by arbitrary stimulation from the eel’s discharge. This suggests that crayfish are more resistant to electric discharges (from Catania, 2015c, reproduced with permission).

What benefit does this behavior provide the eel? Although it intensifies the electric field through prey, a large electric eel would seem to have enough power from just the positive pole. This appeared to be the case when an eel was offered goldfish. But when an electric eel was offered large crayfish, the eel’s initial attack sometimes failed and the crayfish executed the appropriate escape response (Figure 8). Clearly, some prey are more resistive to electricity than others. Curling provides a mechanism for electrifying prey that are both physically and electrically, more resistive. Still, what is the ultimate function of the eel’s curling behavior? The answer seems obvious in retrospect.

### The Induction of Involuntary Fatigue

Recall that high-voltage EOD’s from electric eels activate motor neuron efferents, and hence muscles in nearby prey. In the pithed-fish preparation, this was measured based on whole-body fish tension. Crayfish provide a different window into this effect because many of their paired muscles are asymmetric: the muscles that close their claws are more powerful than the muscles that open them. As a result, the effect of repeated high-voltage volleys from the eel electrifying a crayfish was readily apparent. Unlike the situation in fish, where contraction of symmetric muscle groups resulted in total immobility, in crayfish it was possible to watch the claws open and close with repeated high voltage volleys (see video in Catania, 2015c).

This observation emphasizes an outcome worth re-emphasizing in the context of the eel’s curling behavior. The high-voltage EOD’s result in one-to-one activation of prey motor neurons, causing repeated, high rates of muscle contraction in the captured prey. The eel’s curling strategy is, therefore, a recipe for quickly fatiguing prey muscles. Indeed, the same procedure is used (with a stimulator) in muscle physiology labs to investigate fatigue.

**Figure 9:**
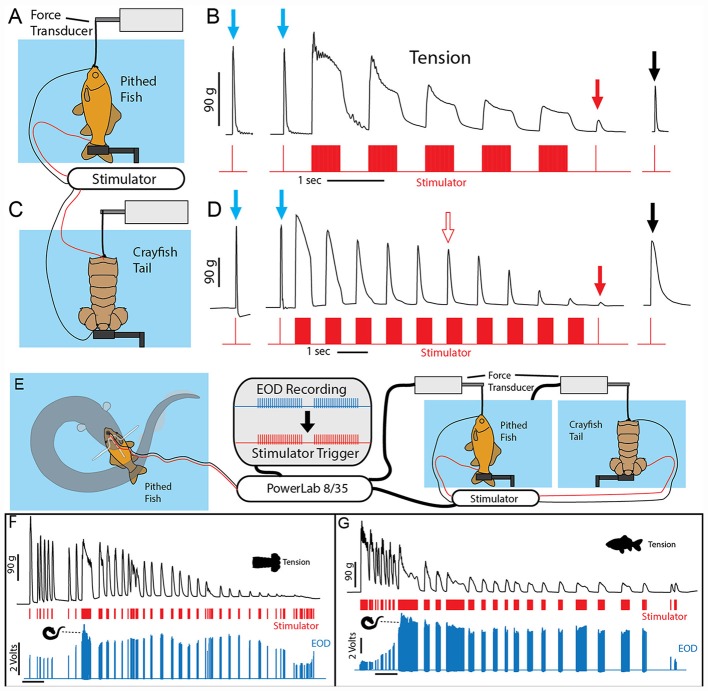
Paradigm used to simulate the effect of eel volleys on prey muscles. **(A)** Pithed fish attached to a force transducer and stimulator. **(B)** Example of whole fish tension responses to single stimulator pulses prior to (blue arrows) a series of 500 ms, 100 Hz volleys, and after (red and black arrows) volleys. Note the dramatic reduction on contractile force following five volleys (red arrow). **(C)** Crayfish tail preparation and stimulator. **(D)** Example of crayfish tail tension responses as described above. Note the difference in time scale, and that more volleys (10) were required to cause a similar reduction in contractile force. **(E)** An electric eel was induced to perform a curling attack on prey-electrode preparation. The recorded high-voltage EOD triggered an SD 9 grass stimulator connected to either a pithed fish preparation, or a crayfish tail preparation connected in turn to a force transducer. **(F)** Tension, stimulator output, and electric eel EOD’s were simultaneously recorded (muscle preparation in adjacent aquarium). Tension in each preparation dropped dramatically over time **(F,G)** and particularly quickly when subjected to the continuous high-frequency stimulation that co-occurs with curling (from Catania, 2015c, reproduced with permission).

To investigate this outcome a stimulator was first used to mimic the effects of an electric eel on prey muscle preparations (Figures 9A–C). Muscle tension from a single stimulator pulse was first measured. This was followed by five bouts of electrical stimulation, each lasting half a second and consisting of 1 ms electrical pulses delivered at 100 Hz. Half a second after the last stimulation bout, muscle tension was then measured (again) for a single stimulator pulse. In a pithed fish preparation, the muscle tension response had dropped drastically. In a crayfish tail preparation, there was only a slight drop in muscle tension after five bouts of stimulation. However, after extending the number of stimulation bouts to 10 (Figure 9D), tension responses in the crayfish tail responses had also dropped drastically. Finally, after a 30 s recovery period, the muscle preparations showed substantial recovery.

These experiments demonstrate the predictable, fatiguing effect of repeated bouts of high-frequency muscle stimulation. The half-second, post-bout testing time for muscle fatigue was chosen because after electric eels engage in this form of behavior while curled, they then reposition the prey for swallowing within a half-second. Thus they need only cause a short period of muscle inactivation to reposition and swallow helpless prey.

To provide more data regarding the effect of eel curling behavior, an additional more elaborate experiment was designed. In this case, the stimulator was configured to be driven by an eel’s high voltage EOD while the eel curled around the previously described fish-electrode preparation (Figures 9E–G). Thus this paradigm tested the effect of the actual, real-time rate of the high-voltage volley on the muscle preparations (i.e., the eel’s EOD drove the stimulator). These cases provided a more realistic view of how eel’s induce fatigue over time. As in the previously described paradigm, the repeated bouts of stimulation resulted in a rapid and drastic reduction in muscle contractile force.

Finally, although this was not explicitly investigated for eels, the oral region of most animals is very sensitive. Electric eels are holding the prey in their mouth while they engage in the curling behavior, so there is every reason to suggest the eel can monitor the contractile force of the prey’s muscles during the curl. This would explain, for example, why eels sometimes electrify the crayfish, while in the curled position, for over a minute (Catania, 2015c). This is far longer than previously observed for any other prey. By the end of such a bout, the crayfish limbs are invariably completely flaccid, and the eel can swallow its prey at leisure.

To summarize these results, electric eels have a strategy for inactivating the muscles of difficult, struggling prey that have been grasped but not subdued. In these cases, the eels concentrate the electric field by sandwiching the prey between the two poles of their long electric organ. This likely ensures activation of the motor neuron efferents in prey that might have more resistive skin or cutical or in the case of juvenile eels, prey might simply not be affected by the output of their weaker electric organ in a linear configuration. Once curled to amplify the local field through the prey, the eels give off repeated volleys. The resulting effect on prey muscles is remarkably similar to the application of a paralyzing agent, such as curare, that blocks the neuromuscular junction. There is a precipitous drop in muscle function. In essence, the eels have a new method for inactivating muscles, through the induction of involuntary fatigue. The strategy is analogous to the use of paralyzing venom, but it takes effect more rapidly.

## Self Defense by the Electric Eel

### Humbolt’s Fish Story

In March of 1800, Alexander von Humboldt supposedly observed an extraordinary encounter between electric eels and horses. He had been traveling in South America and one of his goals was to experiment with electric eels. The first eels that fishermen had brought to him had been poisoned with plant roots and they were “much enfeebled” and useless for experiments (von Humboldt, 1807). Later he encountered a group of locals at the village of El Rastro, and they offered to collect eels by “fishing with horses.” The ensuing battle between the horses and eels is one of Humboldt’s most famous stories of adventure and it has been recounted and illustrated many times in the last 200 years. Most histories of electric fish include an illustration and description of the event. But not everyone believed the story (Catania, 2016). On the other hand, there was no obvious reason for anyone to investigate further. The story had little relevance to the biology of electric eels and it served as an amusing anecdote. It, therefore, came as some surprise when the author discovered a dramatic defensive behavior by electric eels, supporting Humboldt’s account.

### The Leaping Attack

In the course of many of the experimental investigations described above, electric eels were transferred from a home cage to an experimental cage. Depending on the size of the eel, sometimes the net had a metal rim and handle. Although this may not seem wise, the investigator always wore rubber gloves, such that the composition of the handle was inconsequential (or so it seemed). On many occasions, when the metal net was brought toward a large eel, the eel transitioned from a retreat to an explosive attack targeting the metal part of the net. The eel rapidly approached, followed the metal rim to where it exited the water, and then leaped upward while pressing its lower jaw to the metal handle. In coordination with the upward leap, the eel gave off long volleys of its high-voltage EOD. The behavior was particularly surprising because at no other time were electric eels observed leaping upward from the aquarium. Moreover, the unexpected leap was clearly directed toward the metal handle, and therefore coincidently toward the investigator’s hand. Although the rubber glove afforded protection from the eel’s EOD, it was easy to imagine the consequences had there been no glove. The behavior gave the impression of a formidable, electrical attack.

### Measuring the Potential of Leaping Eels

**Figure 10:**
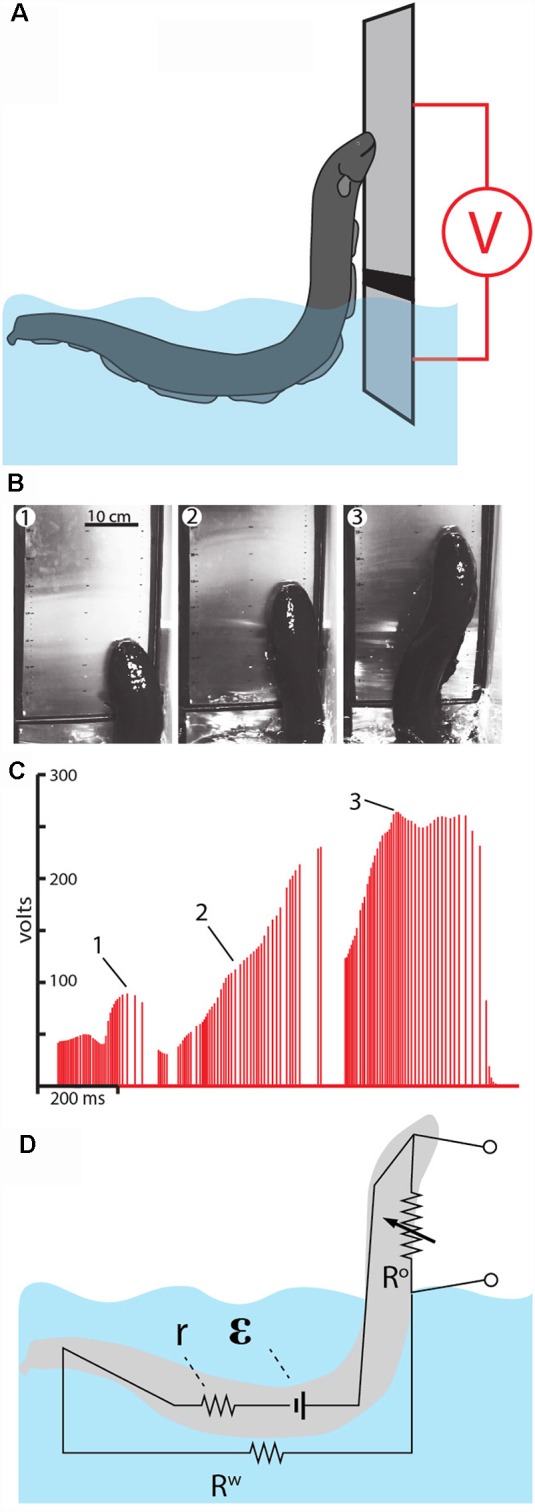
Measurement of voltage during eel leaping defense. **(A)** Schematic of the plate arrangement and voltmeter used to measure the electrical potential as eels ascended the conductor. Black line indicates a non-conductor separating the plates. **(B)** Frames from high-speed video for a shocking leap. **(C)** Voltage measured as the eel ascended. Numbers 1–3 correspond the plates illustrated in **(B)**, indicating the location of the eel at time of discharge. **(D)** The proposed equivalent circuit that develops as the eel emerges from the water. The electromotive force (EMF) of the electrocytes is represented by ε. The resistors include water resistance, the eel’s internal resistance (*r*) and the variable resistor (R°) that represents the current path on, or through, the eel back to the main body of water. This path becomes more resistant as the eel ascends to greater heights (from Catania, 2017a, reproduced with permission).

As was the case for experiments measuring the effect of curling, it was possible to leverage the eel’s behavior to further investigate this novel attack. This was accomplished using two flat metal plates attached to a plastic handle. The lower plate was submerged most of the way in the water, separated from the upper plate (which was entirely above the water) by a thin insulator. A voltmeter was then connected between the two plates. When the eels attacked the apparatus, they emerged from the water pressing their lower jaw against the lower plate while giving off their high-voltage volleys. As they rose higher, they crossed from the lower plate to the upper plate, and thus variations in the electrical potential could be recorded as the eels ascended (Figure 10).

As might be predicted, the electrical potential (voltage) increased dramatically as the eels ascended to greater heights. This is best appreciated by considering the equivalent circuit that is thought to develop (Figure 10D). When fully submerged in the water, the eel’s discharges form an approximately dipole electric field around the eel. In this case, the resistances in the circuit include the internal resistance of the eel (r) and the resistance of the surrounding water (Rw). When the eel emerges from the water and presses its lower jaw against an object, the circuit changes such that a new resistance exists above the water. This is the return path to the water along the eel’s head and upper body (and perhaps through the eel’s body). As the eel ascends to greater heights, the resistance of the return path along the eel increases, hence the measured voltage increases in proportion to height.

Another way to think about this dynamic is to consider the flow of electricity being “pushed” by the eel’s electrocytes. The increasing resistance of the return path along the eel means that more current would be “pushed” through the target (if the target was an animal, rather than a high-impedance voltmeter). In other words, the higher the eel leaps, the less pleasant the experience for the target.

**Figure 11:**
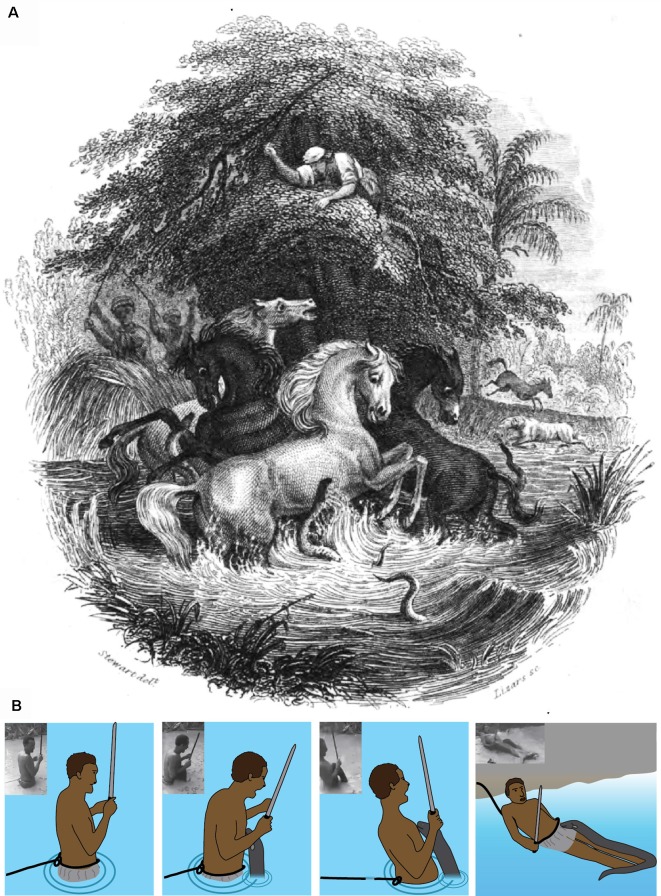
Fishing with horses. **(A)** This illustration depicts the battle between eels and horses observed by Alexander von Humboldt in March of 1800. It was published in 1843 as the front-piece for The Naturalist Library, Ichthyology, Volume V, Part II, the Fishes of Guiana, authored by Robert H. Schomburgk, a friend and protégé of Humboldt’s. **(B)** Schematic and plates showing a fisherman being shocked by an electric eel (Plate **A** is in the public domain, **(B)** from Catania, 2017b, reproduced with permission).

The experiment and observations described above seem to support Humboldt’s story. Yet it was not entirely clear how similar the behavior described above might be to what Humboldt observed. He reported that the eels emerged from the mud and attacked, with at least some eels pressing themselves against the horses (von Humboldt, 1807). But he did not describe eels as leaping out of the water. Since the first observation of eels leaping in the laboratory, two additional pieces of evidence emerged that, combined with the observations described above, further support Humboldt’s story. The first piece of evidence is historical, and comes from a friend of Humboldt’s, as described below and illustrated in Figure 11A.

### Robert Schomburk’s Illustration

Humboldt’s story of the horses and eels has been recounted in numerous publications and books since he first published his own account in 1807 (von Humboldt, 1807). His original publication did not include an illustration of the events, but many subsequent authors provided their own illustrations. The most significant illustration seems to have been lesser known and the least circulated and re-published. This is the front-piece to The Naturalist Library, Ichthyology, Volume V, Part II, the fishes of Guiana, authored by Robert Schomburgk (Schomburgk, 1843). This particular image stands out for two reasons. First, it is by far the most accurate depiction of the events described by Humboldt. Humboldt described fishermen waving reeds, a fisherman that had climbed an overhanging tree above the pool, horses that had escaped, and horses that had collapsed on the nearby shoreline. All of these details are included in the image.

The second reason for its significance is the author. Robert Schomburgk was a friend and admirer of Humboldt (Schomburgk, 1838). Humboldt helped the Schomburgk brothers obtain funding for their own trip to South America (Payne, 2007) and provided advice (Roth, 1922). Given that they knew each other and were in communication about South American travels in the years prior to the image’s production (Roth, 1922) it is possible that Humboldt provided some of his own input for the illustration. Moreover, the illustration shows an electric eel that has emerged from the water to press its lower jaw against one of the horses. There is a remarkable similarity between the eel’s behavior depicted in the image and the behavior observed in recent laboratory experiments (Catania, 2016).

### A Leaping Attack in the Field

The second piece of evidence that also supports Humboldt’s account from 1,800 is the circulation of a very recent video showing a fisherman being attacked by an electric eel. Figure 11B documents this event, which can be viewed from Hawkin (2016). Much can be inferred from the circumstances surrounding this incident. For example, the fisherman wades into a relatively shallow pool while attached to a rope, the other end of which is held by one of his comrades on shore. The fisherman also holds a machete, which is a common means of killing electric eels. The man searches for the eel, but the eel finds him first. The result is a leaping attack onto the man’s chest. The predictable effect is instant paralysis from involuntary muscle activation, as previously described for prey. This possibility was obviously anticipated, as evidenced by the rope, which was used to immediately drag the incapacitated fisherman to shore. The man recovered quickly and the eel (which pursued him to shore) was then killed with a machete. The incident supports Humboldt’s account because it clearly shows that some electric eels in the wild go on the offensive when a potential predator (a large, partially submerged conductor) enters their territory—as occurred with the horses.

## The Equivalent Circuit

### Electromotive Force (EMF), Internal Resistance (r), and Water Resistance (Rw)

When an eel emerges from the water to make direct contact with a potential threat, the circuit that develops is comparatively simple. It was, therefore, possible to investigate most of the variables in the circuit and to estimate how current would flow through different elements (for these measurements and calculations, all values refer to the peak voltages and currents during the high-voltage EOD). The analysis begins with a determination of the (EMF in volts) and internal resistance (r) for each eel. These variables are unique for any given eel at a particular stage of development. As the eel grows and adds electrocytes, its internal resistance and EMF change (the former decreasing, and the latter increasing). Previous investigations of eels (Brown, 1950) and other electric fish (Bell et al., 1976; Caputi et al., 1989; Baffa and Côrrea, 1992) have shown that the electrocytes can be analyzed with methods commonly used for batteries.

One way this can be done is to measure the current (I) that flows for a “short circuited” eel and then measure voltage (V) directly from the skin of an eel that has been removed from the water. Then the resistance (r) can be determined from Ohm’s law (r = V/I). But a more accurate method is to add a variable resistor to the circuit and measure V and I (during each high-voltage EOD) as the resistance is varied. When this is done, a plot of V vs. I yields a straight line with a slope equivalent to the negative of the internal resistance (r). The details of the method are given in Catania (2017a). Using this procedure (Catania, 2017a, b), the EMF and internal resistance (r) of five different electric eels of different sizes were recently measured. Once these variables were determined for each eel, it was then possible to design experiments to measure, for any given eel, the approximate water resistance in the circuit, and subsequently, the resistance of the return path from the eel’s head to water when it leaped to attack. These determinations require only Ohm’s Law, Kirchhoff’s Voltage Law, and algebra in conjunction with various voltage and current measurements. Because these details may not interest all readers, they are omitted for brevity but can be reviewed in Catania (2017a).

**Figure 12:**
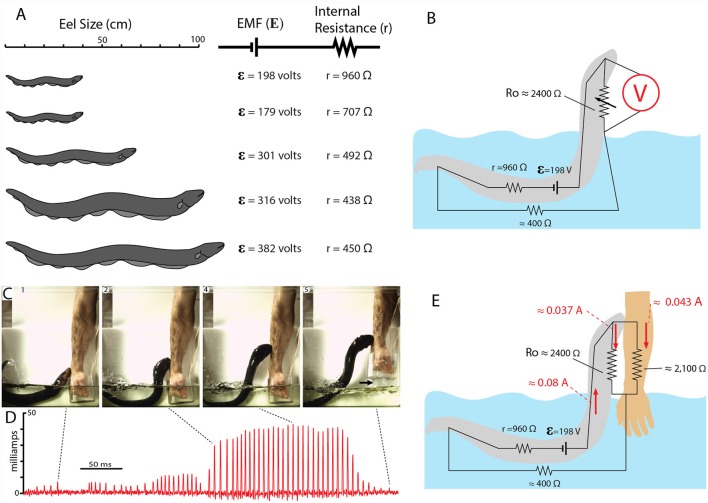
Summary of electromotive force (EMF; ε) internal resistance (*r*), for five different eels and the circuit for the leaping attack. **(A)** Size of each eel in relationship to measured ɛ and *r*. **(B)** Estimate resistances and the maximum resistance of the return path to the water during the leap by eel shown at the top in (**A**; from data in Catania, 2017b). **(C)** Frames from high-speed video documenting the eel and the subject’s arm. Arrow marks break in circuit as arm was withdrawn. **(D)** Current recording during the eel’s shocking leap. Current increased as the eel ascended, as predicted from the equivalent circuit in **(B)**. Current peaks were approximately 43 milliamps. **(E)** Resistances and currents for each component of the circuit during the eel’s leaping attack on a human arm. Resistances are shown in black, currents are shown in red (plates from Catania, 2017a, b; Copyright K.C. Catania).

Figure 12 shows the EMF and internal resistance that were determined for five different eels. The circuit in Figure 12B shows the additional resistance of the return path from head to water (Ro). This configuration is often called a voltage divider circuit, and it has many parallels with circuits used to modulate the amplitude of an electric output in a wide range of electrical equipment. In essence, the eel’s leaping behavior increases the value of the variable resistor, Ro, in proportion to leap height, thus turning up the “volume” of its attack. This comparison to a volume control knob is useful for considering how the eel’s behavior likely evolved. There is no need to imagine a “hopeful monster” scenario in which an ancestral eel suddenly evolved the behavior in one, metaphorical leap. Rather, each successive approximation of the behavior in an ancestor, starting with an approach to the threat in the water, and followed by direct contact, and then by emergence from the water to greater and greater heights (all while giving off the high-voltage EOD), would provide a selective advantage for deterring a predator.

## Completing the Circuit

Many insights can be gained about the dynamics of the electric circuit from the parameters shown in Figure 12B. Yet this configuration of resistors for the leaping attack is incomplete because it lacks the resistance of the target. This, in turn, demonstrates a fundamental problem in circuit analysis. Namely, the total current flowing in a circuit is dependent upon the total resistance of the circuit. In the case of the leaping eel, once a target is added, there are two resistors in parallel above the water. Determining their equivalent resistance, and hence the total circuit resistance is required to calculate total current in the circuit. Having spent considerable effort to determine each of the other variables in this circuit, the story seemed incomplete without this final variable.

Target resistance was therefore determined using the small eel illustrated in Figure 12A (top eel) and a single human subject’s arm. To determine this resistance a device was designed that allowed for measurement of the current through the arm during the small eel’s leaping attack. This consisted of a plastic, non-conductive water chamber with a handle. The inside of the water chamber had an area covered with conductive aluminum tape, but not in direct contact with the subject’s hand. The outside front portion of the chamber was likewise covered with aluminum tape (but electrically isolated from the inner portion of the chamber by virtue of the plastic container’s walls). The inner and outer portions of aluminum tape were then connected with a copper wire through which current could be measured as the eel made its attack. It was then possible to calculate target resistance based on the measured current (Catania, 2017b). The target (arm) resistance was found to be approximately 2,100 ohms. Of course, other eel targets will have other resistances. Nevertheless, this experiment provided important information by indicating whether the eel-target interface or the target-water interface made a substantial contribution to the circuit. The results suggest they did not, instead, the target resistance was in line with predictions (Catania, 2017a). Finally, this final piece of data, in conjunction with the other circuit components, provides a starting point for similar calculations that can be made for different eels attacking different targets in water with more or less resistance.

## Summary

The results of these recent investigations show that electric eels have behaviors that are far more sophisticated than previously thought (at least by the author). The conception of this species as a “one trick pony”—having a powerful weapon that provided the advantage of brute force, without the need for complex behaviors, could not be further from the truth. As is often the case, this seems obvious in retrospect when one considers the eel’s anatomy and physiology in an evolutionary context. Clearly, the electric eel has been strongly selected for electrical power. Electrocytes make up an astonishing proportion of its long body. But there is more than one way to increase power. The first is to add electrocytes. The second is to apply the power from existing electrocytes more efficiently. The second option is arguably less costly than expending many resources to develop, maintain, and power more electrocytes. The curling behavior provides the most obvious example—an eel can literally double the power communicated to prey by simply reorienting its tail. Consider the difference in “cost” between evolving this behavior or, alternatively, doubling the number of electrocytes. A similar argument can be made for the leaping defense. In short, it seems inevitable the strong selection for electrical power would act on both physiology and behavior.

In addition to revealing a number of new behaviors, these studies raise many additional questions for future study. Perhaps the most obvious questions relate to other strongly electric species. Are electric rays and catfish activating the motor neurons in nearby prey? If so, do they use the same strategies for inducing or arresting prey movement? Belbenoit and Bauer (1972) suggested the EOD of the *Torpedo marorata* might serve to startle prey. Similarly, Belbenoit et al. (1979) recorded EOD of hunting electric catfish (*Malapterurus electricus*) and identified frequent pre-volley activity; the investigators specifically suggested these might serve to startle immobile prey. If so, these would be remarkable examples of convergent hunting strategies. Do other strongly electric species also use high-voltage for active electroreception? What are the parallels, from the perspective of sensory processing, between the high rate of an eel’s attack volley and the high rate of a bat’s feeding buzz while echolocating? What kinds of electroreceptors might mediate sensory transduction of high-voltage? What is the composition of an eel’s diet in the wild? Do electric eels specifically target other electric fish (Stoddard, 2002) as suggested from observations of Westby (1988)? How well cloaked are electric fish EOD’s as a result of pressure from eels and other electroreceptive predators (Stoddard, 1999; Stoddard and Markham, 2008)? How does the high skin resistance of an electric fish impact the hunting strategy of an eel? Does active electroreception during a high-speed strike allow tracking precision that cannot be obtained with vision or the mechanosensory lateral line? What role does mechanosensory feedback play as the eel induces involuntary fatigue while curling? How does an eel protect its own nervous system from the high-voltage EOD? These are just a few of the many questions that remain to be investigated.

## Author Contributions

The author confirms being the sole contributor of this work and has approved it for publication.

## Conflict of Interest Statement

The author declares that the research was conducted in the absence of any commercial or financial relationships that could be construed as a potential conflict of interest.
